# External Validation of the IOTA Classification in Women with Ovarian Masses Suspected to Be Endometrioma

**DOI:** 10.3390/jcm10132971

**Published:** 2021-07-01

**Authors:** Lee Cohen Ben-Meir, Roy Mashiach, Vered H. Eisenberg

**Affiliations:** 1 Endometriosis Center of Excellence, Department of Obstetrics and Gynecology, Sheba Medical Center, Ramat-Gan 5262000, Israel; Leebenmeir@gmail.com; 2 Department of Gynecology, Sheba Medical Center, Ramat-Gan 5262000, Israel; Roy.Mashiach@sheba.health.gov.il

**Keywords:** endometriosis, endometrioma, ovarian cyst, IOTA classification, external validation, transvaginal ultrasound, IDEA classification

## Abstract

The study aimed to perform external validation of the International Ovarian Tumor Analysis (IOTA) classification of adnexal masses as benign or malignant in women with suspected endometrioma. A retrospective study including women referred to an endometriosis tertiary referral center for dedicated transvaginal ultrasound (TVUS). Adnexal masses were evaluated using the IOTA classification simple descriptors, simple rules and expert opinion. The reference standard was definitive histology after mass removal at laparoscopy. In total, 621 women were evaluated and divided into four groups: endometrioma on TVUS and confirmed on surgery (Group 1 = 181), endometrioma on TVUS but other benign cysts on surgery (Group 2 = 9), other cysts on TVUS but endometrioma on surgery (Group 3 = 2), masses classified as other findings or suspicious for malignancy on TVUS and confirmed on surgery (Group 4 = 5 potentially malignant, 11 benign). This gave a sensitivity 98.9%, specificity 64%, positive 95.3% and negative 88.9% predictive values, positive 2.74 and negative 0.02 likelihood ratios and 94.7% overall accuracy. The surgical diagnosis for the five masses suspected to be malignant was: borderline serous tumor (2), borderline mucinous tumor (2), and endometrioid lesion with complex hyperplasia without atypia (1). The conclusions were that the IOTA classification simple descriptors, simple rules and expert opinion performs well for classifying adnexal masses suspected to be endometrioma. The most common potentially malignant masses in these women were borderline ovarian tumors.

## 1. Introduction

Transvaginal ultrasound (TVUS) is the first-line imaging technique for evaluation of adnexal masses. The importance of characterizing adnexal masses as benign or malignant is crucial for decision making and subsequent invasive or non-invasive management [1,2,3]. However, standardization of classification has always been a challenge in this field [4,5]. To overcome subjectivity and professional experience of TVUS operators, the International Ovarian Tumor Analysis (IOTA) Group developed a system of standardization in characterization of adnexal masses. A set of “simple descriptors” and “simple rules” characteristics were described and validated in order to introduce simplicity, reproducibility and ease of use. Using the combination of IOTA’s two steps has been shown to yield high conclusive results when determining whether a mass is benign or malignant prior to surgery [6,7,8,9,10].

TVUS plays a significant role in non-invasive investigation in endometriosis patients who do not undergo surgery [11,12]. Endometriosis is a common gynecological disease, with a prevalence of 30–50% in symptomatic women [13] and 1–2% in low-risk populations [14]. Over fifty percent of women with deep infiltrating endometriosis (DIE) present with an endometrioma (ovarian endometriotic cyst) and are referred for high level ultrasound evaluation. Endometrioma is usually a benign adnexal mass, however previous studies have described endometrioma as a risk factor for ovarian cancer [15,16], With the most common histological cancer types being clear cell and endometrioid types [17,18,19,20,21].

The aim of this study was to perform an external validation of the IOTA classification in the evaluation of endometrioma as benign or malignant findings, using TVUS performed during tertiary evaluation of endometriosis patients.

## 2. Materials and Methods

### 2.1. Study Design and Participants

This was a retrospective cohort study in an endometriosis tertiary referral center at the Sheba Medical Center in Tel Hashomer, a center of excellence open for referrals from the entire country, between May 2011–August 2017. Women were referred due to symptoms suggestive of endometriosis (such as severe dysmenorrhea, chronic pelvic pain, dyspareunia, infertility, gastrointestinal and urinary complaints) and suspicious TVUS findings (such as adnexal masses, adenomyosis, deep infiltrative lesions, etc.). The study included women above 18 years of age, who could undergo TVUS, for whom we had complete clinical, sonographic, surgical reports and pathological results. Electronic hospital records and outpatient referral files were used to extract patient information, which included demographic data (age, body mass index, marital status, parity, smoking habits), clinical history (previous cesarean sections, surgical history for endometriosis, fertility treatment history), clinical symptoms (dysmenorrhea, dyspareunia, pelvic pain, infertility, urinary and gastrointestinal symptoms), TVUS findings, and surgical and pathology reports. Patients were followed until September 2020.

### 2.2. TVUS Evaluation of Adnexal Masses

All TVUS scans were carried out using a 7.5 MHz transvaginal probe (Voluson GE Medical Systems, Villach, Austria). The examinations were performed by expert physician sonographers, fully trained gynecologists with more than ten years’ experience in this field (Level-III examiners). Grey scale and color Doppler ultrasound were used to obtain morphological and blood flow variables to characterize each adnexal mass. The examination included a thorough evaluation of all pelvic viscera without bowel preparation. IDEA’s (International Deep Endometriosis Analysis group) systemic approach of sonographic evaluation of endometriosis was taken into consideration in the TVUS examinations following their publication [22]; Endometriosis was diagnosed based on the presence of deeply infiltrative endometriotic (DIE) nodules (for instance uterosacral ligament, bowel and bladder nodules), signs of pelvic adhesions (kissing ovaries or absent sliding of viscera, intestinal adhesions), presence of ovarian endometrioma or overt tubal disease as previously described [23,24,25,26,27,28,29,30,31]. 

Adnexal masses were evaluated using the etc. IOTA classification [4,6]. An endometrioma was diagnosed using simple descriptors as a unilocular tumor with ground glass echogenicity. When the mass did not classify as an endometrioma, the IOTA simple rules [8] were used in order to predict malignancy: (1) irregular solid tumor; (2) presence of ascites; (3) at least four papillary structures; (4) irregular multilocular–solid tumor with a largest diameter of at least 100 mm; and (5) very high color content on color Doppler examination. When at least one of the malignancy features were present and without benign sonographic features: ((1) unilocular cyst; (2) largest diameter of the largest solid component <7 mm; (3) acoustic shadows; (4) smooth multilocular tumor with largest diameter <100 mm and (5) absence of color flow on Doppler examination) we classified the mass as malignant. Benignity of a mass was classified if at least one of the benign features was present, and in the absence of malignancy features [9]. When the IOTA simple rules did not allow to reach a diagnosis (e.g., both malignant and benignity features were present or absence), expert subjective assessment was taken into consideration. In cases of bilateral adnexal mass, the largest or most complex cyst was used for analysis.

### 2.3. Laparoscopic Surgeries

All surgeries were carried out by highly qualified surgeons who specialized in laparoscopic surgery for endometriosis. The indications for surgery were: severe unmanageable symptoms, medical treatment failure, infertility and repeated IVF failure, ovarian cysts larger than 4 cm that was detected on TVUS and DIE involvement of other organs such as the ureter, bladder, rectum, or colon. Patients who wished to conceive and suffered from severe symptoms, or had findings on pelvic examination and/or US evaluation, and/or infertility, were counseled to have surgery before attempting to achieve pregnancy. Other aspects in the decision-making process were age, previous surgery, and ovarian reserve. Yet, the final decision whether to undergo surgery or continue medical or conservative treatment depended on patient preference. 

Comparison between the TVUS examination and the surgical reports of each patient was performed, with the reference standard being definitive histology after mass removal. The patients were allocated into four groups based on diagnosis of endometrioma on TVUS and confirmation or contradiction on surgery and pathological report. Many of the patients presented with a suspected endometrioma, since this is a center that specializes in endometriosis diagnosis and treatment. 

### 2.4. Ethical Aspects

Ethical approval was obtained from the local research ethics committee (IRB). Written informed consent was not required since the ultrasound assessment was offered as part of standard clinical care at the center. No procedure was performed specifically for the purpose of the study and no identifying information is included in the data presented here.

### 2.5. Statistical Analysis

Categorical variables were described as numbers and percentages. Continuous variables were evaluated for normal distribution using histograms and Q-Q plots. Normally distributed continuous variables (such as age) were reported as means ± SD’s and non-normally distributed variables were reported as medians and IQR. The diagnostic performance was estimated by calculating the sensitivity, specificity, positive and negative predictive values, positive and negative likelihood ratios and overall accuracy, with their corresponding 95% CI’s. All statistical analyses were 2-tailed and statistical significance in all tests was set at *p* < 0.05. Statistical analysis was performed using SPSS software (IBM SPSS statistics version 25, IBM Corporation, Armonk, NY, USA, 2017).

## 3. Results

### 3.1. Participants and Clinical Data

**Figure 1:**
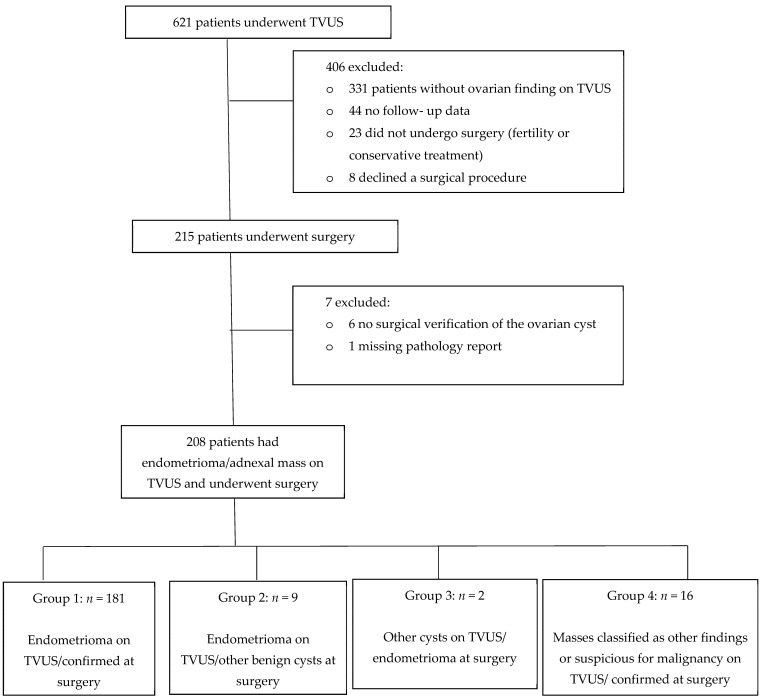
Study flowchart. TVUS (transvaginal ultrasound).

Six hundred and twenty-one women were evaluated for endometriosis and underwent TVUS examination, of whom 331 patients had no adnexal finding and were excluded from the statistical analysis. We further excluded 44 patients for whom we did not have complete follow-up data and 31 women who did not undergo surgery at our center. Two hundred and fifteen women underwent surgery, seven of whom were excluded (six patients had no surgical verification of the ovarian cyst and one with a missing pathology report), leaving 208 women who had an adnexal mass on TVUS and underwent surgery at our center (see Figure 1). Demographic, clinical data and prevalence of TVUS findings are presented in Table 1. Women presented with symptoms of endometriosis including dysmenorrhea in 177 women (85.1%), dyspareunia in 103 (49.5%), gastrointestinal complaints in 96 (46.2%) and urinary complaints in 47 (22.65%). Out of 134 women (64.4%) who tried to conceive, 88 (65.6%) suffered from subfertility. All women were premenopausal. Overall follow-up was on average 32.75 (±25.94) months.

### 3.2. TVUS Evaluation of Adnexal Mass and Surgery Results

Patients were allocated into 4 groups based on a comparison between TVUS findings and surgery results: (1) endometrioma on TVUS which was confirmed on surgery (Group 1, *n* = 181); (2) endometrioma on TVUS but other benign cysts at surgery (Group 2, *n* = 9); (3) other cysts on TVUS and endometrioma at surgery (Group 3, *n* = 2); (4) masses classified as other findings or suspicious for malignancy on TVUS and confirmed at surgery (Group 4, *n*= 16). 

In group 2 the pathological results were: 5 follicular luteinizing cysts, 2 hemorrhagic corpus luteal cysts, 1 serous cystadenofibroma. One of the patients had an inconclusive pathology result, the differential diagnosis was: epidermal inclusion cyst, benign cystic Brenner tumor and/or benign mucinous cyst with extensive squamous metaplasia.

In group 3, two women presented with clear cysts on TVUS which were found to be endometrioma on pathology. Both of the patients had extensive endometriosis with adenomyosis and DIE. One of the patients had bilateral clear cysts on TVUS with a maximal diameter of 27 mm on the left ovary, while on surgery and pathology an endometrioma was confirmed. For the second patient, both TVUS and surgery misdiagnosed a clear cyst (23 mm clear cyst without flow was evaluated on TVUS), while pathology confirmed an endometrioma. 

**Figure 2:**
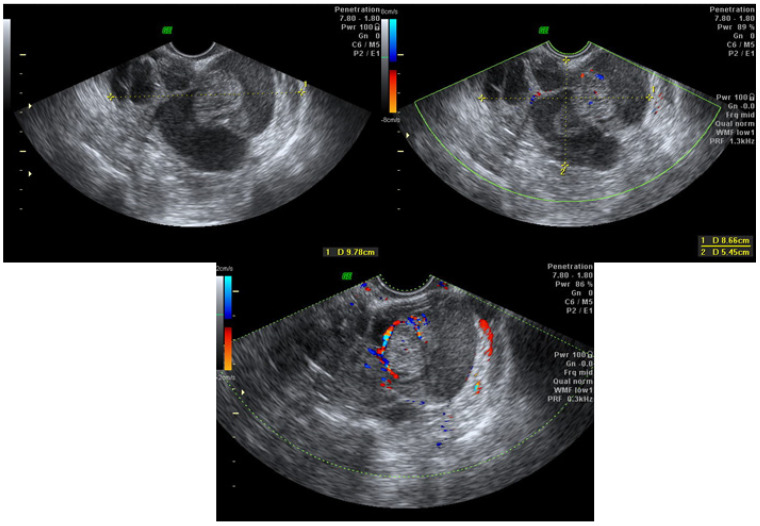
Case number 1; 46 years old woman, presented with dysmenorrhea, infertility (11 cycles of IVF), 1 parity. CA125 = 30. On TVUS multilocular cyst with high suspicious of malignancy, hystological result was borderline mucinous tumor.

**Figure 3:**
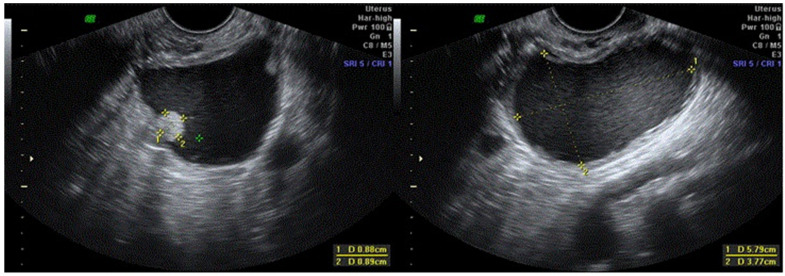
Case number 3; 37 years old woman, presented with dysmenorrhea, infertility (13 cycles of IVF), 0 parity. On TVUS unilocular solid cyst, hystological result was a borderline serous tumor.

**Figure 4:**
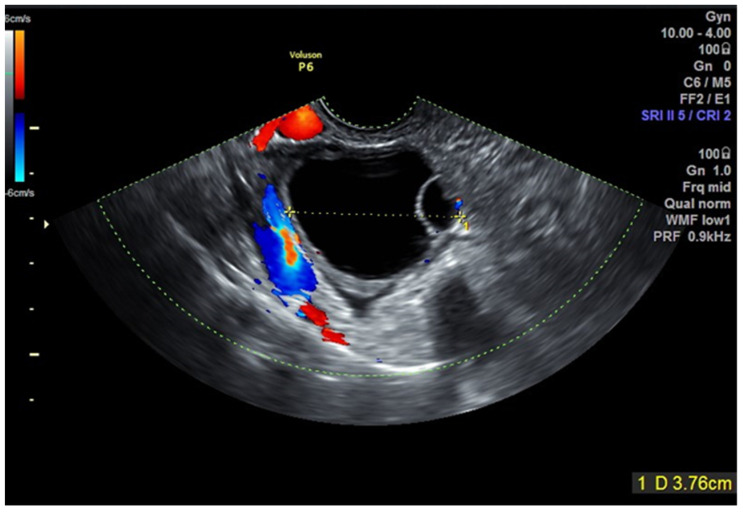
Case number 7; 36 years old woman, presented without symptoms. On TVUS multilocular cyst, hystological result was a serous cystadenoma.

**Figure 5:**
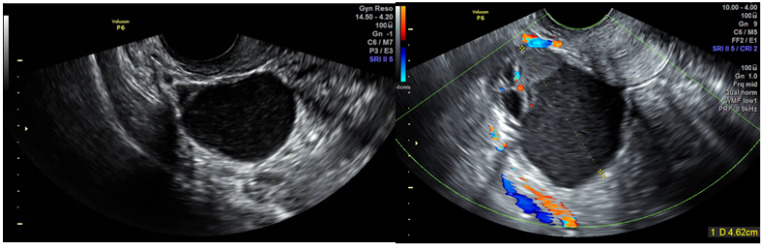
Case number 8; 30 years old woman, presented without symptoms. On TVUS multilocular cyst, hystological result was a mucinous cystadenoma.

Clinical data, TVUS and surgical findings and pathology results of group 4 are presented in Table 2. Out of six masses suspected to be malignant, five were potentially malignant on pathology (two borderline serous tumor, two borderline mucinous tumor and one endometrioid lesion with complex hyperplasia without atypia) (Figure 2, Figure 3, Figure 4 and Figure 5, Videos S1–S3 in Supplementary Materials). The benign mass was a hydro salpinx.

The results of the comparison between TVUS evaluation and pathology results of the cysts were as follows: sensitivity 98.9% (95% CI, 96.11–99.87%), specificity 64% (95% CI, 45.52–82.03%), positive 95.3% (95% CI, 92.26–97.14%) and negative 88.9% (95% CI, 66.15–97.04%) predictive values, positive 2.74 (95% CI, 1.63–4.63) and negative 0.02 (95% CI, 0.00–0.07) likelihood ratios, and 94.7% (95% CI, 90.73–97.33%) overall accuracy.

## 4. Discussion

In this study we performed an external validation of the IOTA classification of adnexal masses in the evaluation of masses suspected to be endometriomas in women referred for dedicated endometriosis TVUS. We found a very high sensitivity and positive predicted values, confirming that the IOTA criteria are an excellent tool for non-invasive diagnoses of adnexal masses in endometriosis patients. 

In the original IOTA study, as in our study, ultrasound examinations were performed by expert sonographers and obtained a sensitivity and a specificity of 92% [6]. Eighty-one percent of 1938 masses were diagnosed successfully using the simple descriptors and simple rules approach. In subsequent studies, external validations have been reported. A study in the U.K. by Sayaneh et al. [32] assessed 301 women with adnexal masses who underwent surgery and reported that 89% of the masses could be classified using IOTA’s simple descriptors and simple rules together. Similar to our study, the reported sensitivity was high, however the specificity was higher than ours, both 95%. Adding the examiners’ subjective assessment, in case the mass could not be fully assessed, reduced the sensitivity and specificity to 93% and 92%, respectively. It is of note that the sonographers in their study were not experts in the field (i.e., Level II examiners), which may explain the higher measured sensitivity in our study (98.9%) compared to that of the original study. 

Testa et al. [33] conducted a multicentric study of 2403 women in 18 centers and six European countries, externally validating the IOTA strategy. Using simple descriptors and simple rules allowed correct classification of 81% of the masses, with a sensitivity and specificity of 95.7% and 73.6%, respectively. Adding a subjective examiner assessment gave a comparable sensitivity of 92.5% and raised the specificity to 87.6%. Similar to our study the most common benign diagnoses were endometrioma (14.3%) and serous cystadenoma (10.8%). Additionally, all ultrasound examinations were performed by level-III examiners. Alcazar et al. [34] reported external validation using the three-step strategy of IOTA with a high sensitivity (94.3%) and specificity (94.9%). In their study, non-expert examiners took expert subjective opinion into account when the mass classification was inconclusive. The study was conducted in two tertiary-care hospitals and included 362 women. 

A small percentage of the masses (4.3%) was misdiagnosed as endometrioma, while other benign cysts were found at surgery (group 2). Our results are similar to the original IOTA study that suggested sonographic characterization for diagnosis of endometrioma [35]. In their study, 66 of 652 (10.1%) masses were misdiagnosed as endometrioma by an expert subjective assessment. A meta-analysis describing the diagnostic accuracy of TVUS for endometrioma [36], showed that most of the cysts, falsely thought to be endometrioma, were in fact hemorrhagic cysts, dermoid cysts, simple cysts, or cystadenomas. The results are not surprising considering the fact that most acute hemorrhagic cysts (as hemorrhagic corpus luteal cysts) have the same ultrasound morphology as an endometrioma, not to mention, ground glass appearance of cyst fluid can be present in different benign masses. Even the most expert of examiners sometimes can be misled by the clinical presentation. In group 4, the IOTA simple descriptors and simple rules for malignancy performed as expected. We confirmed the vast majority of the cysts thought to be malignant. In previous studies, [32,33,34] the false-negative cases were borderline tumors, while in our study, all of those tumors were accurately detected. We had one false-positive case of hydrosalpinx. The description of a multilocular solid cyst with pappillations misled the examiner to suspect a malignancy.

The main strengths of our study are the validation by experienced gynecologic examiners, familiar with the IOTA classification strategy of adnexal masses. Our study used the best reference standard (i.e., histological results), and the ultrasound examinations, surgeries, and histological analysis were conducted in one center, which was important for uniformity of diagnostic methods, laparoscopic procedures, and accuracy of patient follow-up. 

Our study has several limitations which must be addressed: The fact that we are a tertiary referral center for endometriosis evaluation and treatment means that we see a high-risk population, most of whom do have endometriosis. The relatively low specificity that was found can be attributed to observer bias. However, to minimize this bias, we applied IOTA’s strict protocol for the evaluation of all adnexal masses. Additionally, using IOTA’s simple rules method by non-expert examiners does in fact yield a higher specificity compared to that reached by expert examiners [37].

Another limitation is that the simple descriptors, simple rules, and expert’s subjective assessment were assessed simultaneously by experienced sonographers. As a result, a comparison between the different steps of the IOTA strategies was difficult. Moreover, comparing the impact of expert and non-expert examiners during endometriosis evaluation using the IOTA strategy may be beneficial to reducing the time delay to diagnosis for these patients.

## 5. Conclusions

The IOTA classification simple descriptors, simple rules and expert opinion performs well for classifying adnexal masses suspected to be endometrioma, in women evaluated for endometriosis in a tertiary referral center. The most common potentially malignant masses in this population were borderline ovarian tumors. Operators who perform endometriosis diagnosis can use the IOTA classification when evaluating masses that they encounter during the examination. Additionally, the clinicians referring the patient can use the IOTA classification for diagnosis and need not wait for the tertiary exam for evaluation of the mass. It seems that teaching clinical operators the IOTA classification along with the IDEA consensus will benefit patients being referred for endometriosis evaluation.

## Figures and Tables

**Table 1:** Demographic, clinical data and prevalence of TVUS findings of patients attending an endometriosis tertiary referral center and included in the study (*n* = 208).

Parameter	Group 1 (*n* = 181)	Group 2 (*n* = 9)	Group 3 (*n* = 2)	Group 4 (*n* = 16)	Total (*n* = 208)
Age, mean ± SD, years	33.6 ± 6.1	30.4 ± 10.4	43 ± 1.4	34.1 ± 7.2	33.6 ± 6.5
BMI, median (IQR)	22.5 (19.9–25.6)	22.3 (20.7–22.8)	23	23.4 (20.6–26.6)	22.5 (20.1–25.5)
Smoking (%)	56 (30.9%)	4 (44.4%)	1 (50%)	6 (37.5%)	67 (32.2%)
Nulliparous (%)	115 (63.5%)	5 (55.6%)	0	10 (62.5%)	130 (62.5%)
Previous Cesarean section (%)	17 (9.4%)	1 (11.1%)	0	2 (12.5%)	20 (9.6%)
Previous surgery (%)	74 (40.9%)	4 (44.4%)	1 (50%)	10 (62.5%)	89 (42.8%)
**Additional TVUS findings (%)**					
Maximal size of cyst, median (IQR), mm	50 (41–66)	44.5 (39.5–53)	40	46 (38.5–64.7)	49 (40.5–65.5)
Kissing ovaries	53 (29.8%)	2 (22.2%)	0	3 (18.8%)	58 (28.3%)
Uterosacral ligaments nodule	85 (47.2%)	5 (55.6%)	0	2 (12.5%)	92 (44.4%)
Retro cervical nodule	46 (25.4%)	0	0	0	46 (22.1%)
Recto sigmoid nodule	57 (31.5%)	3 (33.3%)	0	2 (12.5%)	62 (29.8%)
Bladder nodule	8 (4.4%)	0	0	1 (6.3%)	9 (4.3%)
Intestinal nodule	26 (14.4%)	1 (11.1%)	0	1 (6.3%)	28 (13.5%)
Pouch of Douglas obliteration	85 (47%)	4 (44.4%)	0	3 (18.8%)	92 (44.2%)

SD—standard deviation; BMI—body mass index; TVUS—transvaginal ultrasound; IQR—interquartile range.

**Table 2:** Clinical data, findings on TVUS and surgery and pathology results of women with malignant adnexal masses and different benign adnexal masses.

Case Number	Age (Years)	Parity	Side of Finding	Maximal Size of Finding (mm)	TVUS * Description	Suspected Malignancy	Additional Findings	Pathology Result of Adnexal Mass	Follow-Up (Months)	Comments
1	46	1	Left	98	Multilocular cystic	Yes	Uterus myomatosus, right endometrioma, rectosigmoid nodule	Borderline mucinous tumor	53	Normal follow-up, no recurrence.
2	32	2	Right	100	Multilocular cystic	Yes	None	Borderline mucinous tumor	5	Normal follow-up, no recurrence.
3	37	0	Left	58	Unilocular solid	Yes	None	Borderline serous tumor	92	Recurrence after 4 years, underwent USO **, normal follow-up.
4	28	0	Right	40	Unilocular cystic low-level echogenicity	Yes	None	Borderline serous tumor	54	Normal follow-up, no recurrence. Spontaneous pregnancy.
5	36	0	Right	47	Unilocular cystic low-level echogenicity	Yes	Rectovaginal nodule	Endometrioid lesion (complex hyperplasia without atypia)	34	Normal follow-up. Infertility-repeated IVF cycles, no pregnancy.
6	22	0	Left	46	Multilocular solid cyst with papillations	Yes	Uterosacral ligament nodules	Hydrosalpinx	22	Normal follow-up, no recurrence.
7	36	3	Left	45	Multilocular cystic	No	Uterosacral ligament nodules	Serous cystadenoma	15	Normal follow-up, no recurrence.
8	30	0	Right	66	Multilocular cystic	No	None	Mucinous cystadenoma	31	Recurrence after 2.5 years, underwent cystectomy for the third time, normal follow-up.
9	38	0	Left	33	Unilocular cystic low-level echogenicity	No	Deep lesion around right ureter	Mucinous cystadenoma	45	Normal follow-up, no recurrence. Conceived twice with fertility treatments.
10	32	0	Left	98	Unilocular cystic	No	None	Mucinous cystadenoma	19	Normal follow-up, no recurrence. Spontaneous pregnancy.
11	40	3	Left	61	Unilocular solid	No	None	Fibro-thecoma	4	Normal follow-up, no recurrence.
12	24	0	Right	53	Multilocular cystic	No	Bladder nodule, left endometrioma.	Multi-cystic benign mesothelioma with decidual changes	11	Normal follow-up, no recurrence.
13	39	2	Right	20	Unilocular solid	No	Bladder nodule, uterosacral ligament nodule	Mature cystic teratoma	25	Normal follow-up, no recurrence.
14	43	1	Right	74	Multilocular cystic	No	Intestinal adhesions	Hemorrhagic corpus luteum	5	Normal follow-up, no recurrence.
15	40	2	Left	35	Unilocular cystic	No	Uterosacral ligament nodule	Hemorrhagic corpus luteum	78	Normal follow-up, no recurrence.
16	23	0	Left	69	Unilocular cystic low-level echogenicity with papillation (para ovarian)	No	None	Para-ovarian cyst: Serous Cyst adenofibroma	31	Normal follow-up, no recurrence. Spontaneous pregnancy.

* TVUS—transvaginal ultrasound; ** USO—unilateral salpingo-oophorectomy.

## Data Availability

Not applicable.

## Supplementary Materials

The following are available online at https://www.mdpi.com/article/10.3390/jcm10132971/s1, Video S1: case number 7—serous cystadenoma, Video S2: case number 8—mucinous cystadenoma, Video S3: case number 10—mucinous cystadenoma.
